# Early recognition of growth abnormalities permitting early intervention

**DOI:** 10.1111/apa.12266

**Published:** 2013-05-13

**Authors:** Morey Haymond, Anne-Marie Kappelgaard, Paul Czernichow, Beverly MK Biller, Koji Takano, Wieland Kiess, on behalf of the participants in the global advisory panel meeting on the effects of growth hormone

**Affiliations:** 1 Children's Nutrition Research Center, Baylor College of MedicineHouston, TX, USA; 2 Novo Nordisk A/SBagsværd, Denmark; 3 Hôpital Necker Enfants MaladesParis, France; 4 Massachusetts General HospitalBoston, MA, USA; 5 University of TokyoTokyo, Japan; 6 Hospital for Children and Adolescents, University of LeipzigLeipzig, Germany

**Keywords:** Growth failure, Growth hormone, Short stature

## Abstract

Normal growth is a sign of good health. Monitoring for growth disturbances is fundamental to children's health care. Early detection and diagnosis of the causes of short stature allows management of underlying medical conditions, optimizing attainment of good health and normal adult height.

**Conclusion:**

This review summarizes currently available information on monitoring for short stature in children and conditions usually associated with short stature and summarizes the authors’ conclusions on the early recognition of growth disorders.

## Introduction

**Figure 1:**
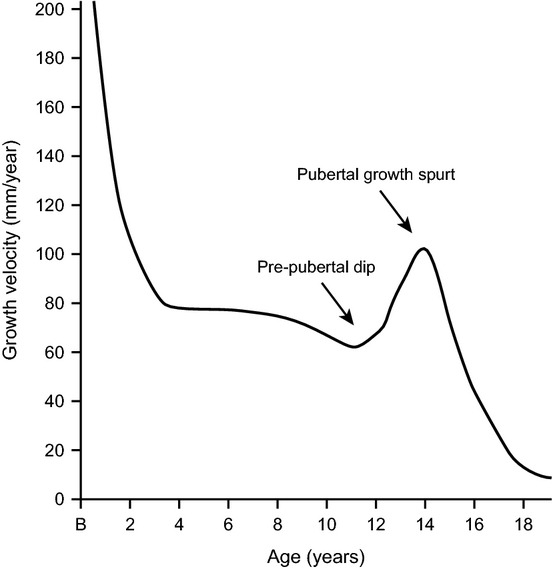
A typical growth velocity curve. Growth is fast in early childhood and then slows down until the start of the pubertal growth spurt.

Assessment of a child's height and weight is one of the best indicators of his or her general health and well-being. Abnormal growth might indicate the existence of underlying disease in the apparently normal child. Early detection and diagnosis of short stature minimizes the impact of any underlying health condition and optimizes final adult height. However, short stature in children is frequently unrecognized in early childhood and thus diagnosed at a late age, which decreases the opportunity to intervene and improve both their health outcomes and stature 1. A child's actual height or length results from initial length at birth and the rate (or velocity) of growth over time. Growth velocity is highest at birth and progressively decreases until the pubertal growth spurt causes the adolescent increase in height with sudden deceleration to a growth velocity of 0 as epiphyseal fusion occurs (Fig. 1). Genetic (familial) short stature (where a child can inherit decreased final height from their parents) is amongst the most common causes of short adult stature 2,3. Other common causes of short adult height include constitutional delay of growth and puberty (in which statural growth falls below 5% of the growth curve after 1–2 years of age and is further delayed due to late start to puberty), nutritional deficiency, precocious puberty, dysymorphic syndromes (or genetic diseases), endocrine diseases or hormonal problems, systemic illness or psychosocial deprivation. The shorter the child at the time of recognition, the more likely it is that the child is not growing normally 4.

## Why is it Important to Recognize Growth Failure as Early as Possible?

Early detection of abnormal growth, and identification of the underlying cause(s), is critical for appropriate treatment. In many cases, poor growth may be the earliest sign of a medical problem. Normal growth is the result of a complex interaction between genetic, hormonal and environmental/nutritional factors. Correcting the pathologic conditions associated with short stature will usually result in normalization of growth. Endocrine disorders are rarely the cause of short stature, but when present are highly treatable, so are especially important to diagnose early. The possibility for proper treatment depends both on the early identification of these children and on appropriate evaluation by knowledgeable clinicians.

**Key notes**

- Normal growth is a sign of good health in children.
- Monitoring growth allows early detection of the causes of poor growth.
- Early recognition of poor growth allows early intervention optimizing the possibility of achieving good health and a normal adult height.

## Definition and Assessment of Short Stature

### Definition of short stature and growth retardation

By definition, normal growth encompasses the 95% confidence interval (CI) for a specific population. Most children who have a normal growth pattern but remain below the lower 2.5 percentile (approximately −2.0 standard deviations [SD]) are otherwise normal. The further below −2.0 SD (2.5 percentile) an individual's growth falls, the more likely it is that there is a pathological condition keeping him or her from achieving their genetically determined height potential. Growth retardation refers to a downward deflection of the growth velocity with the resultant growth curve crossing the SD lines or percentiles.

### Assessment of short stature

Any assessment of height needs to be normalized relative to the population that the individual is from and a definition of short stature made in reference to that population 5. A diagnosis of short or tall stature is usually based on a child's height measurement lying outside ± 2 SD on a growth chart; however, exact cut-off points may vary between country and between growth charts. In the UK, height below the 0.4th centile is accepted as a screening test for short stature 6. In the Netherlands, severe short stature is defined as height standard deviation score (SDS) < −2.5 SDS 7. Current growth charts have been derived using data collected in large population samples of normal healthy children 8. Many countries have developed growth reference charts that are specific to their populations for use in routine clinical practice 9,10.

There is considerable debate regarding whether a single measurement of height at school entry is the best way to identify growth-related disorders, or whether it is better to monitor growth over time (growth velocity).

**Figure 2:**
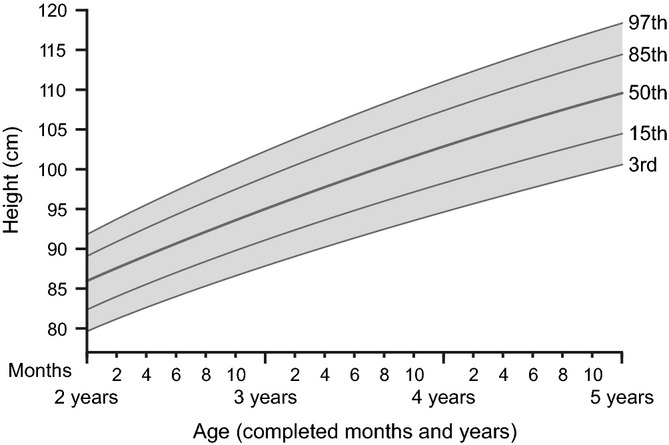
Length-for-age percentiles for girls, from 2 to 5 years. The 50th percentile line (the population average) shows that 50% of normal girls have height (or stature) shorter than the line and 50% are taller. At the lowest line (5th percentile), only 5% of the population of girls/boys are shorter. The growth curves were constructed using data from the WHO Child Growth Study, World Health Organization, Geneva, 2006.

Importantly, a single height measurement only identifies children whose height is outside the normal range. In contrast, repeated height measurements over time allow for calculation of a growth rate (or growth velocity) and can be used to define abnormal growth in terms of a crossing of the height centiles, thereby identifying abnormality through the pattern of growth within the individual 11 (Fig. 2). Growth velocity provides a superior measure because changes in actual height only become evident after altered growth rates have been sustained for a period of time. A normal child tends to follow a given centile line or pattern; deviations in growth away from the percentile are difficult to detect over short intervals using the growth curve; this is one of the most important reasons for calculating the growth rate or growth velocity 12. Evaluating the growth velocity at each routine and acute illness visit provides the earliest identification of problems with growth.

Growth velocity is normal if growth is maintained along an isobar line. When growth slows and crosses the height centiles, even if still within the normal range on the growth chart, a pathological aetiology is more likely. Growth deceleration is defined as a growth velocity that is below the 5th percentile for age and gender (e.g. <5 cm/year after the age of 5 years) or a height drop across two or more percentiles on the growth chart. When growth velocity is abnormally decreased, height measurements will progressively fall across isobars, sometimes termed ‘falling off the curve’. Conversely, acceleration of growth velocity results in crossing the upper isobars but not all growth acceleration is beneficial; exposure to abnormal sex steroid whether from an exogenous or endogenous source can lead to early accelerated growth, premature fusion of the epiphyses and ultimately, short adult stature. Growth velocity must therefore be interpreted in conjunction with attained height because position on the growth curve is essential to interpreting the growth rate and determining the effectiveness of interventions.

The intrinsic biological complexity of the dynamics of human growth, however, makes the use and interpretation of measurements of growth challenging. Growth velocities of individual children are characterized by very high variability in consecutive growth intervals. Intermittent short growth arrests and growth spurts are frequent in child development. It is not unusual for a child to grow at the 95th velocity centile during one month and at the 20th velocity centile in the following month. Correlations between subsequent different patterns of growth are generally low; this reflects both a natural pattern of saltatory growth and possible catch-up or catch-down growth that contributes to overall narrowly canalized patterns in the attained growth trajectories of individual children.

Growth velocity is best assessed using measurements taken at 3- to 4-monthly intervals in infants and at 6-monthly intervals in older children. As young children often have growth spurts, the most accurate estimates of yearly growth velocity are derived from averaging 12-monthly height (or length) measurements as compared with averaging measurements from intervals of < 12 months. Although the latter approach may intuitively appear more useful, imprecision in height measurements means that, in the short term, height velocity may not adequately identify reduced growth during routine growth monitoring 13. Although obtaining and plotting height measurements does not require expensive or sophisticated equipment, training and attention to detail are warranted 14. Indeed, two of the most common reasons for the misdiagnosis of growth disorders and inappropriate referrals for further evaluation are errors in height measurement or inaccurate plotting of values on a growth curve 15. Measuring children's height is subject to error as a consequence of poor technique, variations between instruments and observers, diurnal variation and plotting mistakes 13. A degree of imprecision is, however, inevitable, as children are not rigid objects and do not have an exact or correct height. Nevertheless, with appropriate training and care, single height measurements can be obtained in community practice with acceptable precision, especially in children who are aged over 3 years 15,16. An appropriate growth chart is an essential tool for the screening, surveillance and monitoring of children's growth.

The growth of preterm children differs from that of children born at full term and is dependent on their gestational age. Evidence suggests that boys, in particular, may be vulnerable to the complications of preterm birth that influence growth 17. When assessing height on a growth chart it is therefore important to correct for premature birth and to use their corrected age, rather than their actual age since birth. For example, a child born at 31 weeks’ gestation was born nine weeks early (40–31 = 9 weeks). The corrected age for this child will always be nine weeks less than his or her actual age since birth.

### Midparental height

The performance of height measurement may be improved if a child's height is corrected for parental height; some children may be incorrectly referred for further investigation if their height potential has not been considered. As adult stature is influenced significantly by genetic factors, a child's adult height can be predicted on the basis of midparental height. The adjusted midparental height (target height) is the potential or genetic target height of a child and can be calculated from the midparental height or, if the height of both parents is not available, from the height of one parent or sibling, and adjusting for the child's sex 18:

Boys: (father's height + mother's height + 13 cm)/2
Girls: (father's height + mother's height − 13 cm)/2

Irrespective of the actual height measurement, a child whose height SDS falls outside the parental target range is more likely to have a growth disorder. However, it has been argued that it is only appropriate to consider midparental height when both parents are of normal stature and the calculation may be misleading in the assessment of short children 19.

## Health Implications of Short Stature

Short stature, or decreasing growth velocity (falling off a normal growth isobar line), is a frequent reason for paediatric consultations. Children and parents have concerns about the possibility of underlying disease as well as the perceived social consequences of short stature, such as teasing or bullying at school or even self-esteem, physical challenges and social relations 20,21.

Prompt identification and effective treatment of medical disorders such as cardiac defects, inflammatory bowel disease, cystic fibrosis, chronic renal failure, undiagnosed or chronic poorly controlled diabetes mellitus and malignancy are important for good health as well as for normalizing growth. Treatment of any hormonal abnormalities associated with short stature is typically associated with other benefits. Children treated for hypothyroidism benefit from improved energy and bowel function after treatment. Children with growth hormone deficiency (GHD) or endogenous Cushing syndrome are usually more energetic and have better muscle function and bone density after treatment.

Popular stereotypes associate short stature with impairments in psychological well-being and functioning compared with those with normal or tall stature 22–24. There is, however, a lively ongoing debate about the nature and degree of such impairments with recent evidence from cross-sectional population studies questioning the association between psychological status and quality of life with height 25–28.

As short stature is a common phenomenon and psychopathology is prevalent in both short- and normal-statured populations, care must be taken to ensure that conclusions are not made relating problems of adjustment in short children with stigmatization. Clearly, further research is warranted to disentangle height-dependent and height-independent psychological dysfunction in short-statured children. For some patients with short stature, there may be additional neuropsychological impairment related to the underlying medical condition that may impact on psychological functioning and adjustment to short stature.

## The Causes of Short Stature in Children

**Figure 3:**
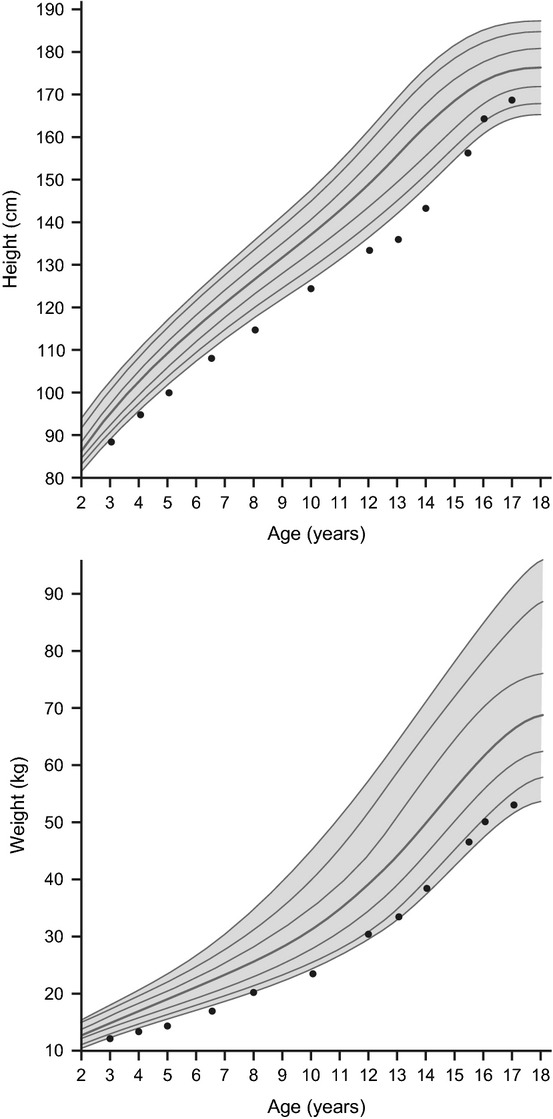
Constitutional growth delay (CGD). Children with CGD, the most common cause of short stature and pubertal delay, typically have retarded linear growth within the first 3 years of life. In this variant of normal growth, linear growth velocity and weight gain slows, beginning as young as 3–6 months, resulting in downward crossing of growth percentiles which often continues until 2–3 years of age. At that time, growth resumes at a normal rate, and these children grow either along the lower growth percentiles or beneath the curve but parallel to it for the remainder of the prepubertal years. At the expected time of puberty, the height of children with CGD begins to drift further from the growth curve because of delay in the onset of the pubertal growth spurt. Catch-up growth, onset of puberty and pubertal growth spurt occur later than average, resulting in normal adult stature and sexual development. Data are for a representative growth curve showing the 3rd, 10th, 25th, 50th, 75th, 90th, 97th percentiles. Dots show a typical growth curve for a child with CGD.

There are a number of conditions that might lead to a reduced growth rate and/or short stature, which potentially could be detected early through growth monitoring. In monitoring for short stature, many children will turn out to be normal variants. These children have no underlying pathology or psychosocial issues causing their reduced height. Some of these children may have constitutional delay in growth and/or puberty, where growth is at a normal rate but bone age is delayed relative to their actual age 29 (Fig. 3). Many have family histories of similar growth patterns, and although many may eventually achieve a normal adult height (but later than their peers), others remain short as adults 30. If the child's parents are also short then the child may be considered to have genetic or familial short stature. If these normal variants of growth are considered unlikely, it is important to investigate further to identify and treat a potentially serious condition initially manifesting as delayed growth. In a school-based study, 14% of children who were shorter than the 3rd percentile and growing at < 5 cm/year had an underlying medical condition. For 5% of children the short stature was endocrine in origin 31.

The large number of clinical conditions associated with short stature can make the task of identifying the cause of short stature challenging. The European Society of Paediatric Endocrinology (ESPE) classified the main causes of short stature into three groups: (i) primary growth disorders, where the condition is intrinsic to the growth plate; (ii) secondary growth disorders, where the milieu of the growth plates change as a consequence of the condition; and (iii) when there is no identifiable cause of short stature (idiopathic short stature [ISS] or growth failure of unknown aetiology) 32. The main causes of growth retardation are shown in Table 1.

**Table 1:** Causes of short stature

*1. Primary growth failure*
Clinically defined syndromes including Down syndrome, Turner syndrome, Noonan syndrome, Prader–Willi syndrome and Silver–Russell syndrome
Small for gestational age (SGA) with failure of catch-up growth
Congenital bone dysplasia, for example, achondroplasia, hypochondroplasia
*2. Secondary growth disorders*
Endocrine causes
Growth hormone deficiency (GHD) either congenital or acquired (hypothalamic-pituitary region lesions such as craniopharyngioma or head trauma)
Multiple pituitary deficiencies
Cushing syndrome
Hypothyroidism
Consequences of precocious puberty
Other disorders of the GH–IGF-I axis
IGF-I deficiency
ALS-deficiency
IGF-I resistance
Metabolic disorders
Poorly controlled diabetes mellitus
Disorders of lipid, carbohydrate, protein metabolism, for example, chronic renal insufficiency
Disorders in organ systems and systemic disorders, for example, cardiac, pulmonary (cystic fibrosis), liver, intestinal (short bowel syndrome and coeliac disease), renal, chronic anaemia, juvenile arthritis
Psychosocial conditions such as emotional deprivation, anorexia nervosa
Systemic or local glucocorticoid therapy
Treatment of childhood malignancy, for example, chemotherapy, total body irradiation

**Figure 4:**
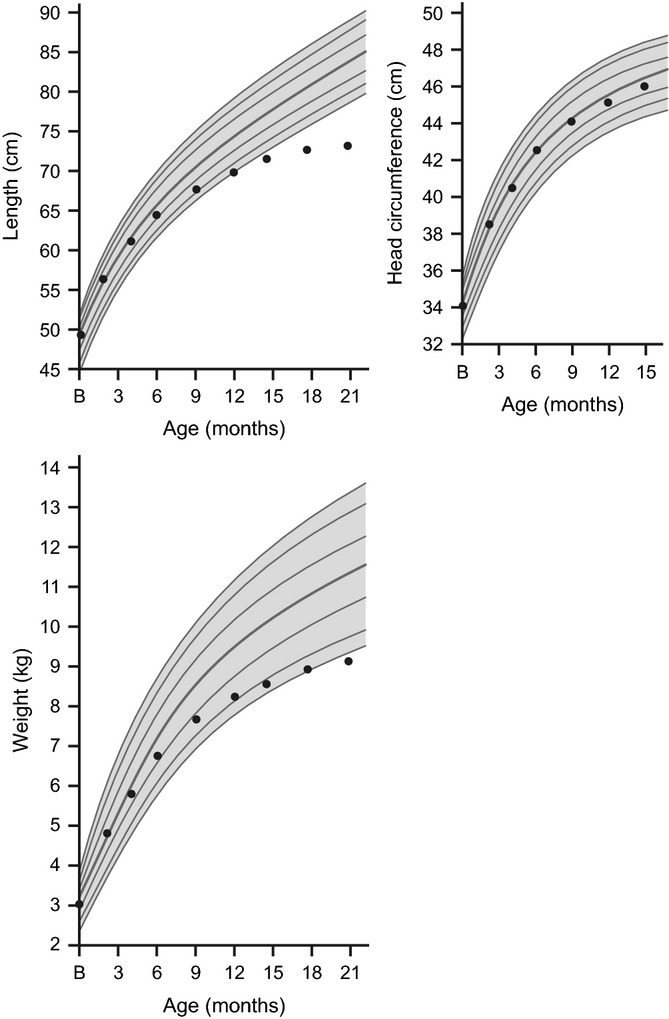
Severe growth hormone deficiency (GHD). If weight and height growth are delayed with a normal head circumference, an endocrinopathy, such as GHD, is suspected. Children with GHD have a slow or flat rate of growth, usually < 2 inches (5 cm) per year. The slow growth may not appear until a child is 2–3 years old. The child will be much shorter than most or all children of the same age and gender. Data are for a representative growth curve showing the 3rd, 10th, 25th, 50th, 75th, 90th, 97th percentiles. Dots show a typical growth curve for a child with GHD.

**Figure 5:**
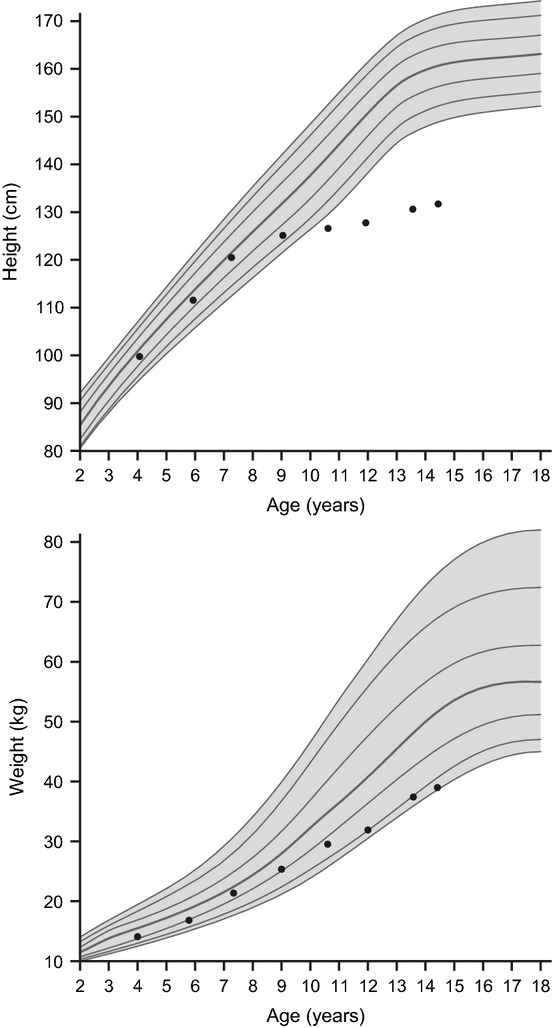
Acquired hypothyroidism. Short stature and decreasing percentiles dating from the onset of hypothyroidism are characteristic of the condition. Data are for a representative growth curve showing the 3rd, 10th, 25th, 50th, 75th, 90th, 97th percentiles. Dots show a typical growth curve for a child with hypothyroidism.

**Figure 6:**
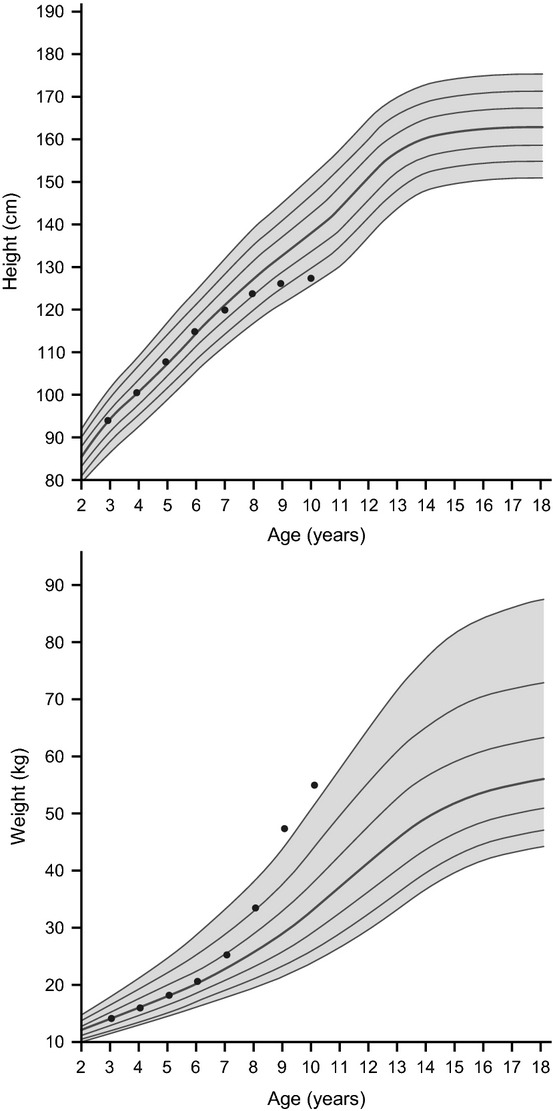
Children with Cushing syndrome characteristically demonstrate an increase in weight velocity with a concomitant decrease in height velocity. Data are for a representative growth curve showing the 3rd, 10th, 25th, 50th, 75th, 90th, 97th percentiles. Dots show a typical growth curve for a child with Cushing syndrome.

A deceleration of linear growth in a well-nourished or obese child may be an indication of an endocrine cause of short stature such as GHD, hypothyroidism or glucocorticoid excess. GHD may be congenital or acquired and may be an isolated deficiency or occur in association with other pituitary hormone deficiencies (Fig. 4). Congenital GHD may occur in patients who suffered perinatal asphyxia or may result from early prenatal embryologic malformations including malformations of the central nervous system (CNS), such as septo-optic dysplasia. Acquired GHD is idiopathic in the majority of diagnoses, but may result from tumours (craniopharngioma, glioma), traumatic head injury, CNS infection or irradiation, or surgical damage to the pituitary or hypothalamus. In these patients, slow growth is the characteristic feature at diagnosis. Whilst congenital hypothyroidism is usually identified by newborn screening, many cases of primary hypothyroidism are acquired later in childhood, usually as an autoimmune condition (Fig. 5). In patients with untreated hypothyroidism of either aetiology, growth velocity is slow, and bone age is delayed relative to chronological age. Glucocorticoid excess is usually iatrogenic, caused by pharmacologic therapy of a concurrent disease such as renal or connective tissue disease or cancer, but sometimes results from excess endogenous glucocorticoid production (Cushing syndrome) because of a tumour (adrenal, ectopic or pituitary). In patients with short stature due to glucocorticoid excess, a slow growth velocity is accompanied by a delayed bone age relative to chronological age, weight gain and elevated blood pressure (Fig. 6). Whether a child exposed to glucocorticoid excess ultimately attains a normal adult height depends on the steroid dose they were exposed to, the duration of exposure, and whether or not the sex steroids have affected skeletal maturation.

Short stature with decreased weight-for-height ratio may indicate the presence of a chronic systemic disease. Indeed, almost all systemic diseases may attenuate growth to a degree that is dependent on the severity and treatment of the underlying disease. In some cases (e.g. irritable bowel syndrome, coeliac disease, chronic renal insufficiency), growth may be impaired for several years before the gastrointestinal or renal symptoms are evident. In contrast to these more subtle diseases, growth failure associated with other chronic disease (e.g. heart, immunological, etc.) is clinically evident and obviously related to the underlying condition. Children who are undernourished or suffer malnutrition also present with similar symptoms; short stature and reduced weight for height. In children with a nutritional deficiency despite access to food (e.g. anorexia nervosa or poorly controlled type 1 diabetes), weight loss is more pronounced than the decline in linear growth, and there may also be delayed sexual maturity and bone age.

Genetic or syndromic causes of short stature are often diagnosed because of abnormalities found during clinical examinations. For example, relatively more shortening of the limbs than the spine may suggest achondroplasia, whereas dysmorphic features of the eyes, ears or facial abnormalities may prompt evaluation for chromosomal disorders such as Turner syndrome or Down syndrome. Some girls with Turner syndrome may, however, have an entirely normal physical appearance, rather than the typical phenotype that includes webbed neck, characteristic facies, short metacarpals, etc. For this reason, a karyotype should always be considered in a short girl. Unfortunately, when the diagnosis of Turner syndrome is sought in a young lady with delayed puberty, the time of optimal treatment for height and other psychological factors will have passed, emphasizing the importance of monitoring growth velocity over time. A change in the rate of growth during early childhood must always be investigated. For some children, growth may be normal until the age of 10–12 years, after which the rate of growth is markedly slowed. If constitutional delay of growth and puberty is the cause of this growth deceleration, it will be followed by a spontaneous acceleration of growth closely associated with the eventual pubertal growth spurt. Unfortunately, this same pattern of growth deceleration after the age of 10–12 years can be associated with a pathologic growth delay. Hence, although such a growth pattern may be benign, careful investigation is required. The late effects of therapy for childhood leukaemia may include endocrine abnormalities, especially deficiencies in growth hormone, gonadotropin and sometimes thyrotropin, all of which may be associated with growth failure. In very young children, cranial–spinal radiotherapy can result in precocious puberty and short adult stature. However, primary hypothyroidism more commonly occurs following treatment for leukaemia if the thyroid gland was within the radiation field. With the increasing numbers of survivors of cancer therapy, it has become important to identify and treat short stature presenting in such cases.

Growth failure without an organic aetiology but associated with behavioural disturbance and psychosocial stress has been termed psychosocial short stature. This condition includes failure to thrive, poor growth secondary to chronic malnutrition and idiopathic hypopituitarism. The wide spectrum of signs and symptoms associated with psychosocial growth failure mean that the condition is difficult to identify with certainty, and there is a substantial risk of underestimating growth disturbances as an indicator of child neglect and abuse 33. Some children show spontaneous catch-up growth when removed from the source of stress, without further treatment 34.

It has been suggested that boys come to medical attention because of short stature more frequently than girls. Male predominance in referrals for short stature has been reported across age groups, which may account for a disparity, often observed, between males and females in the evaluation and treatment of short stature 35–37. Grimberg et al. reported that the number of boys referred for growth retardation was almost double that of girls 36. It is interesting, however, that in the Netherlands, in a study evaluating the Dutch consensus guidelines for the diagnostic work-up of growth failure performed in two hospitals, of the 542 children evaluated, 284 were boys and 258 were girls (male/female ratio 1.1) 7.

## The Incidence of Poor Growth as an Index of Organic Disease

In the Wessex Growth Study, further investigation of 147 children who had been passed as ‘short normal’ at school entry identified eight cases of previously unidentified disease 4. In only four was it remediable (hypothyroidism, coeliac disease, lead poisoning and GHD), but in all cases, it was informative. These eight conditions had been missed at the school entry medical examination. The proportion of children with organic disease increased with the degree of short stature; seven of the 12 children whose heights were more than 3 SDs below the mean had some organic disease.

Thus, 1 of 10 children with a height between −2 and −3 SD have an organic cause of their short stature, whereas when height is below −3 SD, organic cause may be found in up nearly 60% of such children 4. In other studies, an organic cause of short stature has been described in between 0.7 and 4.5% 36,38,39 of the screened population with causes including previously undiagnosed juvenile hypothyroidism and GHD.

Hence, although a proportion of children with short stature referred for further evaluation may be diagnosed with either familial short stature or constitutional delay of growth and puberty, there are children with endocrinological or nonendocrinological pathologies for whom early detection and diagnosis of organic causes of abnormal growth will provide the best chance for good health and a normal adult height.

## Guidelines for Growth Monitoring

The aim of growth monitoring or screening programmes is to identify children with various treatable causes of abnormal growth who have been missed or who failed to present in clinical practice. A review of 31 growth-monitoring studies concluded that a single height screening might identify between 1:545 and 1:1793 new cases of potentially treatable conditions 40. Despite its widespread use, however, the importance of growth monitoring in detecting growth disorders and its impact on child health is not well recognized 41.

Indeed, even if growth monitoring is performed, children with treatable causes of abnormal growth are frequently diagnosed at, or treatment started at, an older age, precluding the opportunity for the child to achieve normal or near normal height 42,43. In some cases, a lack of trained staff may mean that children are unable to get routine growth checks at school 44. Indeed, as recently as 2011, Yardeni et al. showed that adherence to guidelines for evaluation of short stature in the primary clinic referring children to a specialist unit was inadequate; children were frequently referred without important data on previous growth and parental height, and many laboratory tests crucial to the primary care physician's evaluation were lacking 45.

To be effective, a growth monitoring programme must be accurate, continuous and widely used in the population. There is, however, substantial variation in national guidelines for the diagnostic approach to short stature with respect to criteria used, equipment and growth charts 8,46.

In an assessment of Dutch children with Turner syndrome, referral criteria based on absolute height measurement were found to be less useful than criteria that adjusted for midparental height or height velocity 46. In the UK, children are routinely measured for height at all contact points with their family practitioner, up to the point of school entry at 5 years of age; children with the lowest height < 0.4th centile (−2.66 SDS) are referred for further assessment 47. Using the 0.4th centile identifies a population of children with very short stature of whom between one-third and one-half of children have an identifiable growth disorder 48.

An earlier Finnish guideline, still in use today, is based on cut-off limits for height SDS minus target height SDS and a range of cut-off limits for delta height SDS, depending on the age of the child and the length of the age interval 49. No account is taken of midparental height or change in height SDS with time.

An evidence-based guideline for the referral of children with short stature, published in 2008 7, reported a difference in the efficacy and efficiency of referral criteria for young children (below the age of 3 years) and older children. In children < 3 years of age, a low height SDS (height SDS < −3) was shown to be the most useful criteria for detecting short stature. In older children (3–10 years), combined use of ‘short for target height’ (height SDS minus target height SDS < 2 and height SDS < 2) and height SDS < 2.5 as well as height deflection (height SDS decrease > 1.0 SD) provided the best means of identifying children with short stature 50. Using these guidelines in children aged 3–10 years, a distance to target height of > 2 SD combined with height SD of < −2 detected 85.7% of children with Turner syndrome and 76.5% of children who are short with various growth disorders with a low false-positive rate (1.5–2.0).

**Figure 7:**
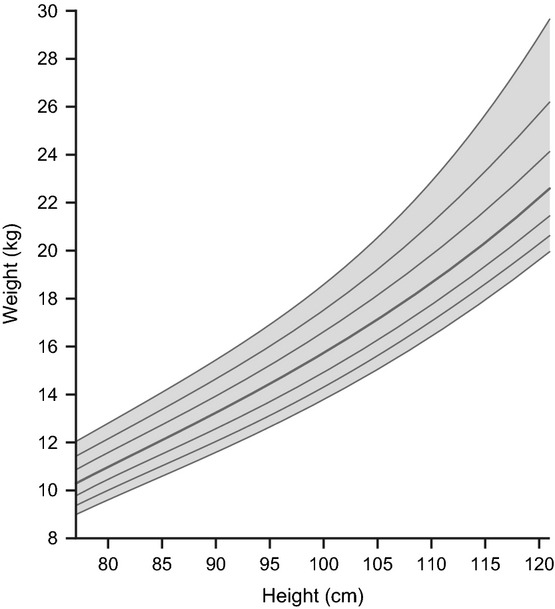
Individual growth chart 3rd, 10th, 25th, 50th, 75th, 90th, 97th percentiles: Boys weight-for-stature. Reproduced from Kuczmarski RJ, Ogden CL, Guo SS et al. 2000 CDC growth charts for the United States: Methods and development. National Center for Health Statistics. *Vital Health Stat* 2002; 11(246).

For a good differential diagnosis, a thorough medical and family history should complement the physical examination to determine signs, symptoms and clues that may indicate a specific disease. Relevant points in the history include birth characteristics, symptoms suggestive of chronic organic diseases, psychiatric diseases and/or severe emotional disturbances. It is also important to determine whether a slow pattern of growth occurred in either parent. The physical examination should include a systematic examination of all body systems including a careful search for dysmorphic features and disproportionate shortening of the limbs. After a thorough medical history and physical examination has been undertaken, analysis of the growth curve and weight-for-height measurements should be done (Fig. 7) 51.

Community growth monitoring programmes do not, however, identify all cases of growth-related conditions. In some cases, children are referred following parental concerns or concerns raised by family practitioners during routine examinations 52.

Patient height and growth pattern are recognized as important drivers of referrals from primary care to specialist endocrinology clinics. In a retrospective evaluation of 9062 patients referred during a 28-year period by their family physician or other paediatricians to two Brisbane children's hospitals, 29% (n = 2599) were referred for short stature 35. Of these, 58% were diagnosed with familial short stature, constitutional delay of growth or were of normal height, but 931 (36% of referrals) had a disease causing short stature, including 12% with Turner syndrome, Noonan syndrome or Russell–Silver syndrome, 6% born small for gestational age (SGA), 8% with diseases in organ systems and 5% with GHD 35. As an organic cause for short stature was found in over one-third of referred cases, it provides a strong indication that all such referrals should be taken seriously.

In less extreme cases of short stature, referrals are sometimes monitored by a ‘wait and see’ policy, using height velocity, often over a very short period, as a secondary screening tool 38. Children apparently growing well can then be dismissed and the rest referred for specialist advice.

Large-scale monitoring programmes can provide detailed information on the best procedures for identifying short stature in the population by providing a systematic method for diagnosing growth disorders in previously undiagnosed children by identifying pathological growth curves as well as identifying secular trends for growth in the population. In 1998, a network was established to collect data on growth input by paediatricians in Germany (>160 currently) 39. Children with heights above the 97th centile or below the 3rd centile of the German synthetic norm curve were highlighted to the relevant practice. Children were then referred for specialist investigation if this was considered necessary. Of 60 984 children assessed, 2775 children (4.5%) had a height less than the 3rd percentile of the normative reference. Amongst the 2775 children referred for specialist follow-up for short stature, there were 38 new cases of GHD (1:1605 children screened), four new cases of Turner syndrome (1:15 246 children screened), two new cases of juvenile hypothyroidism (1:30 492 children screened) and three new cases of psychosocial growth failure (1:20 328 children).

## Conclusions

Failure to grow is an important and key clinical condition for the general practitioner to recognize. Short stature should be identified, diagnosed and treated appropriately and without delay. Normal growth is a sign of good health, but ill children and adolescents often grow slowly, making monitoring for growth disturbances of critical importance in paediatric health care. The principal reason to study abnormal growth in infants and children is to identify conditions that may threaten good health and life. The most useful tests in distinguishing the short normal child from one with a pathologic condition are accurate height measurements over time and calculation of the growth velocity. Most apparently, healthy children who are short but growing at a normal growth velocity are healthy. In contrast, a child whose growth velocity is declining, irrespective of their absolute height, deserves thorough evaluation. Prompt recognition of the cause of short stature, by measuring children early and often, provides the best chance for a child to achieve an ideal health outcome as well as the potential to reach an adult height within the normal population range. In light of our ability to diagnose remediable diseases using accurate growth assessment, the clinical significance of early recognition of short stature is clear.

## Abbreviations

CDG: Constitutional growth delay
CI: Confidence interval
CNS: Central nervous system
GHD: Growth hormone deficiency
ISS: Idiopathic short stature
MPH: Midparental height
SDS: Standard deviation scores
SD: Standard deviation
SGA: Small for gestational age

## References

[1] Thomas M, Massa G, Craen M, de Zegher F, Bourguignon JP, Heinrichs C (2004). Prevalence and demographic features of childhood growth hormone deficiency in Belgium during the period 1986–2001. Eur J Endocrinol.

[2] Sultan M, Afzal M, Qureshi SM, Aziz S, Lutfullah M, Khan SA (2008). Etiology of short stature in children. J Coll Physicians Surg Pak.

[3] Papadimitriou A, Douros K, Papadimitriou DT, Kleanthous K, Karapanou O, Fretzayas A (2012). Characteristics of the short children referred to an academic paediatric endocrine clinic in Greece. J Paediatr Child Health.

[4] Voss LD, Mulligan J, Betts PR, Wilkin TJ (1992). Poor growth in school entrants as an index of organic disease: the Wessex growth study. Br Med J.

[5] Wit JM, Clayton PE, Rogol AD, Savage MO, Saenger PH, Cohen P (2008). Idiopathic short stature: definition, epidemiology, and diagnostic evaluation. Growth Horm IGF Res.

[6] National Screening Committee http://www.screening.nhs.uk/growth.

[7] Grote FK, Oostdijk W, De Muinck Keizer-Schrama SM, van Dommelen P, van Buuren S, Dekker FW (2008). The diagnostic work up of growth failure in secondary health care; an evaluation of consensus guidelines. BMC Pediatr.

[8] de Onis M, Wijnhoven TM, Onyango AW (2004). Worldwide practices in child growth monitoring. J Pediatr.

[9] de Onis M, Onyango A, Borghi E, Siyam A, Blössner M, Lutter C (2012). Worldwide implementation of the WHO Child Growth Standards. Public Health Nutr.

[10] Freeman JV, Cole TJ, Chinn S, Jones PR, White EM, Preece MA (1995). Cross sectional stature and weight reference curves for the UK, 1990. Arch Dis Child.

[11] Rogol AD, Clark PA, Roemmich JN (2000). Growth and pubertal development in children and adolescents: effects of diet and physical activity. Am J Clin Nutr.

[12] Brook CG, Hindmarsh PC, Healy MJ (1986). A better way to detect growth failure. Br Med J (Clin Res Ed).

[13] Voss LD, Wilkin TJ, Bailey BJ, Betts PR (1991). The reliability of height and height velocity in the assessment of growth (the Wessex Growth Study). Arch Dis Child.

[14] Cole TJ (2002). Assessment of growth. Best Pract Res Clin Endocrinol Metab.

[15] Voss LD, Bailey BJ (1994). Equipping the community to measure children's height: the reliability of portable instruments. Arch Dis Child.

[16] Betts PR, Voss LD, Bailey BJR (1992). Measuring the heights of very young children. Br Med J.

[17] Bocca-Tjeertes IF, van Buuren S, Bos AF, Kerstjens JM, Ten Vergert EM, Reijneveld SA (2012). Growth of preterm and full-term children aged 0–4 years: integrating median growth and variability in growth charts. J Pediatr.

[18] Cole TJ (2000). A simple chart to identify non-familial short stature. Arch Dis Child.

[19] Wright CM, Cheetham TD (1999). The strengths and limitations of parental heights as a predictor of attained height. Arch Dis Child.

[20] Molinari E, Sartori A, Ceccarelli A, Marchi S (2002). Psychological and emotional development, intellectual capabilities, and body image in short normal children. J Endocrinol Invest.

[21] Johansen H, Andresen IL, Naess EE, Hagen KB (2007). Health status of adults with short stature: a comparison with the normal population and one well-known chronic disease (rheumatoid arthritis). Orphanet J Rare Dis.

[22] Voss LD (2001). Short normal stature and psychosocial disadvantage: a critical review of the evidence. J Pediatr Endocrinol Metab.

[23] Sandberg DE, Brook AE, Campos SP (1994). Short stature: a psychosocial burden requiring growth hormone therapy?. Pediatrics.

[24] Wheeler PG, Bresnahan K, Shephard BA, Lau J, Balk EM (2004). Short stature and functional impairment: a systematic review. Arch Pediatr Adolesc Med.

[25] Lee JM, Appugliese D, Coleman SM, Kaciroti N, Corwyn RF, Bradley RH (2009). Short stature in a population-based cohort: social, emotional, and behavioral functioning. Pediatrics.

[26] Ross JL, Sandberg DE, Rose SR, Leschek EW, Baron J, Chipman JJ (2004). Psychological adaptation in children with idiopathic short stature treated with growth hormone or placebo. J Clin Endocrinol Metab.

[27] Sandberg DE (2011). Psychosocial aspects of short stature and its management: good deeds require good science. Horm Res Paediatr.

[28] Coste J, Pouchot J, Carel JC (2012). Height and health-related quality of life: a nationwide population study. J Clin Endocrinol Metab.

[29] Spadoni GL, Cianfarani S (2010). Bone age assessment in the workup of children with endocrine disorders. Horm Res Paediatr.

[30] Poyrazoğlu S, Günöz H, Darendeliler F, Saka N, Bundak R, Baş F (2005). Constitutional delay of growth and puberty: from presentation to final height. J Pediatr Endocrinol Metab.

[31] Lindsay R, Feldkamp M, Harris D, Robertson J, Rallison M (1994). Utah Growth Study: growth standards and the prevalence of growth hormone deficiency. J Pediatr.

[32] Wit JM, Ranke M, Kelnar CJH (2007). ESPE classification of paediatric endocrine diagnoses. Horm Res.

[33] Gohlke BC, Khadilkar VV, Skuse D, Stanhope R (1998). Recognition of children with psychosocial short stature: a spectrum of presentation. J Pediatr Endocrinol Metab.

[34] Jagtap VS, Sarathi V, Lila AR, Bukan AP, Bandgar T, Menon P (2012). Hyperphagic short stature: a case report and review of literature. Indian J Endocrinol Metab.

[35] Thomsett MJ (2010). The spectrum of clinical paediatric endocrinology: 28 years of referrals to an individual consultant. J Paediatr Child Health.

[36] Grimberg A, Kutikov JK, Cucchiara AJ (2005). Sex differences in patients referred for evaluation of poor growth. J Pediatr.

[37] Lee JM, Davis MM, Clark SJ, Kemper AR (2007). Threshold of evaluation for short stature in a pediatric endocrine clinic: differences between boys versus girls?. J Pediatr Endocrinol Metab.

[38] Ahmed ML, Allen AD, Sharma A, Macfarlane JA, Dunger DB (1993). Evaluation of a district growth screening programme: the Oxford Growth Study. Arch Dis Child.

[39] Keller E, Gausche R, Meigen C, Keller A, Burmeister J, Kiess W (2002). Auxological computer based network for early detection of disorders of growth and weight attainment. J Pediatr Endocrinol Metab.

[40] Fayter D, Nixon J, Hartley S, Rithalia A, Butler G, Rudolf M (2007). A systematic review of the routine monitoring of growth in children of primary school age to identify growth-related conditions. Health Technol Assess.

[41] Hindmarsh PC, Cole TJ (2004). Height monitoring as a diagnostic test. Arch Dis Child.

[42] Macfarlane A (1995). Epidemiology of short stature due to growth failure. J Med Screen.

[43] American Academy of Pediatrics (2000). Recommendations for Preventive Pediatric Health Care Committee on Practice and Ambulatory Medicine. Pediatrics.

[44] Fry T (2008). If it's worth doing, let's do it!. Arch Dis Child.

[45] Yardeni D, Loewenthal N, Limony Y, Hershkovitz E (2011). Ethnic and gender inequities in the evaluation of referred short children. Horm Res Paediatr.

[46] van Buuren S, van Dommelen P, Zandwijken GR, Grote FK, Wit JM, Verkerk PH (2004). Towards evidence based referral criteria for growth monitoring. Arch Dis Child.

[47] Hall D, Cole T, Elliman D, Gibson P, Logan S, Wales J (2008). Growth monitoring. Arch Dis Child.

[48] Lacey KA, Parkin JM (1974). Causes of short stature. A community study of children in Newcastle upon Tyne. Lancet.

[49] Sorva R (1989). Growth evaluation: parent and child specific height standards. Arch Dis Child.

[50] Oostdijk W, Grote FK (2009). de Muinck Keizer-Schrama SM, Wit JM. Diagnostic approach in children with short stature. Horm Res.

[51] Kuczmarski RJ, Ogden CL, Guo SS, Grummer-Strawn LM, Flegal KM, Mei Z (2002). 2000 CDC Growth Charts for the United States: Methods and Development. National Center for Health Statistics. Vital Health Stat.

[52] Jellinek D, Hall DM (1994). How are children's growth problems diagnosed?. Child Care Health Dev.

